# Proinflammatory versus anti-inflammatory response in sepsis patients: looking at the cytokines

**DOI:** 10.1186/cc14016

**Published:** 2014-12-03

**Authors:** D Anand, S Ray, S Bhargava, S Das, A Garg, S Taneja, D Dhar, L Mohan Srivastava

**Affiliations:** 1 Department of Biochemistry and Critical Care Medicine, Sir Ganga Ram Hospital, New Delhi, India; 2 Department of Critical Care and Emergency Medicine, Sir Ganga Ram Hospital, New Delhi, India

## Introduction

Despite improvements in supportive care, mortality rates in sepsis remain substantially high. Sepsis exhibits phases of enhanced inflammation, alternating with immune suppression with a resultant variant time point of mortality; yet no human study has correlated levels of cytokines to the timeline of mortality. Our study attempts to analyze the association of levels of proinflammatory and anti-inflammatory cytokines in sepsis with the timeline of death in terms of early (<5 days) versus late (>5 days) mortality, and day of death. We also assessed correlation of these cytokines with length of stay.

## Methods

The study protocol was approved by Institutional Ethics Committee. Subjects were 74 consecutive patients with community-acquired severe sepsis/septic shock admitted to the ICU of a tertiary care superspeciality hospital. Blood samples drawn on days 1, 3 and 7 of admission were analysed for proinflammatory cytokine (TNFα) by chemiluminescent immunometric assay and anti-inflammatory cytokine (IL-10) by ELISA. Subjects were segregated on basis of: ratio of proinflammatory and anti-inflammatory mediators on day 1 of admission into patients with predominant proinflammatory or predominant anti-inflammatory response. Survival and time point of mortality into survivor, early mortality and late mortality groups. Statistical analyses were performed using SPSS version 17.

## Results

There were 37 patients each in predominant proinflammatory and predominant anti-inflammatory groups. The number of deaths was 11 and 17 respectively in these groups. However, there was significantly higher early mortality in the proinflammatory group as compared to the anti-inflammatory group (7 vs. 1, *P *= 0.0247). On the contrary, late deaths were significantly higher in the anti-inflammatory group as compared to the proinflammatory (16 vs. 4 *P *= 0.0017). The ratio of proinflammatory/anti-inflammatory cytokines (TNF/IL-10) on day 1 was significantly lower in patients of late death (*n *= 20) as compared to patients of early death (*n *= 8) and survivors (*n *= 46) as shown in Table 1. Further, the median day of death was significantly delayed in patients in the anti-inflammatory as compared to the proinflammatory group (20 vs. 5, *P *< 0.001). Length of hospital stay amongst survivors was significantly longer in the anti-inflammatory as compared to the proinflammatory group (23 vs. 10 *P *< 0.001).

**Table 1:** TNF/IL-10 ratio in study groups at different time points

	Early death (≤ 5 days) (*n *= 8)	Late death (>5 days) (*n *= 20)	Survivors (*n *= 46)	*P *value
Day 1	1.81 (1.00 to 3.44)	0.50 (0.31 to 0.90)	1.22 (0.43 to 3.91)	0.020*
Day 3	1.12 (0.50 to 3.91)	1.01(0.20 to 2.21)	2.5 (0.90 to 3.91)	0.158
Day 7	-	1.25 (0.59 to 2.38)	1.79 (0.75 to 3.90)	0.256

Data presented as median (IQR). Kruskal-Wallis test was performed for significance between three groups. *P < 0.05 considered significant.

## Conclusion

Our preliminary data suggest that in sepsis, the ratio of proinflammatory/anti-inflammatory cytokines on day 1 is significantly associated with time point of mortality; hence, this ratio can be used to particularize management. Further studies are in progress to substantiate the role of proinflammatory and anti-inflammatory cytokines in this subset of patients. Moreover, since predominant anti-inflammatory response was associated with later death, role of immunomodulators in sepsis needs to be explored.

